# Evaluating the Efficacy and Safety of a Nasal Medical Device (NESOSPRAY HE-G) in Acute Rhinosinusitis: A Randomized, Double-Blind, Placebo-Controlled Clinical Trial

**DOI:** 10.3390/medicina62091631

**Published:** 2026-08-25

**Authors:** Marine Delmas, Manon D’Almeida, Séverine Dameron-Puech, Rémi Shrivastava

**Affiliations:** Vitrobio, ZAC de Lavaur, 63500 Issoire, France; m.delmas@vitrobio.com (M.D.); m.d_almeida@vitrobio.com (M.D.); s.dameron@vitrobio.com (S.D.-P.)

**Keywords:** rhinosinusitis, NESOSPRAY HE-G, nasal spray, symptom relief, Nonpharmacological treatment, medical device, glycerol

## Abstract

*Background and Objectives*: Acute rhinosinusitis is associated with a substantial deterioration in quality of life, including symptoms such as nasal obstruction, facial pressure, cough, and sleep disruption. Growing interest in non-pharmacological management strategies has been observed, particularly among populations in whom systemic treatments may be limited, such as pediatric patients and pregnant individuals. NESOSPRAY HE-G is a nasal device containing a filmogenic glycerol formulation designed to create a protective barrier and induce an osmotic effect, thereby promoting nasal decongestion and mucosal protection. *Materials and Methods:* A randomized, double-blind, placebo-controlled study was conducted to assess the performance and safety of NESOSPRAY HE-G (*n* = 20) compared with a saline comparator (*n* = 20) in patients aged 3 years and older presenting with acute rhinosinusitis and a baseline Rhino-Sinusitis Severity Score (RSSS) of at least 25/50. The investigational product was administered as three sprays per nostril, four times daily, over a maximum period of 15 days or until symptom resolution. Efficacy was primarily evaluated through changes in the total RSSS and in key symptoms, including nasal congestion, facial pain, cough, sleep disturbance, and fever. Safety assessments included monitoring adverse events (AE) and evaluating local tolerability. *Results*: Baseline characteristics were comparable between groups. Treatment with NESOSPRAY HE-G resulted in a statistically significant reduction in total RSSS from Day 2 onwards, with sustained effects throughout the study period. Improvements were also observed across individual symptoms, including early relief of nasal congestion and reductions in cough and facial pain, with faster onset compared to the comparator. Sleep quality showed progressive improvement. Resolution of fever occurred in both groups, with an earlier effect observed in the NESOSPRAY HE-G arm. No serious adverse events (SAE) or hypersensitivity reactions were reported. The product demonstrated good tolerability. *Conclusions*: NESOSPRAY HE-G provides rapid and sustained alleviation of symptoms associated with acute rhinosinusitis. Its topical mode of action, based on osmotic activity and barrier formation, represents a suitable non-pharmacological option, particularly for patients seeking alternatives to systemic therapies or presenting contraindications to such treatments.

## 1. Introduction

Rhinosinusitis is a widespread inflammatory disorder affecting both the nasal mucosa and the paranasal sinuses, with a negative impact on patients’ daily functioning and quality of life. Common clinical manifestations include nasal obstruction, facial discomfort or pressure, impaired sleep, and, in some cases, cough [1,2]. Based on disease duration, rhinosinusitis is generally classified as either acute or chronic [3,4]. Acute rhinosinusitis lasts less than 12 weeks and is commonly associated with viral infections or environmental triggers, occasionally progressing to secondary bacterial involvement. Chronic rhinosinusitis (CRS), by contrast, persists for more than 12 weeks, may be associated with nasal polyposis and immune alterations, and is characterized by more complex inflammatory mechanisms often requiring long-term management [5,6,7]. Regardless of these clinical differences, both forms represent a significant burden in terms of healthcare resource utilization and patient quality of life [8].

Acute rhinosinusitis, the focus of this study, remains a common clinical concern. Current epidemiological data suggest that rhinosinusitis affects an estimated 10–15% of the global population, with the majority of cases presenting in an acute form [9]. Among its symptoms, nasal congestion is the most frequently reported, with a prevalence estimated at approximately 30% in the general population [10]. Regardless of its etiology, nasal congestion significantly impairs quality of life through its impact on daily activities, particularly sleep, work or school performance, and social interactions [11].

According to the EPOS 2020 guidelines, the diagnosis of acute rhinosinusitis is primarily symptom-based and relies on the sudden onset of nasal obstruction/congestion and/or nasal discharge associated with facial pain/pressure and/or reduction in or loss of smell. In most uncomplicated cases, diagnosis is established clinically without the need for routine imaging investigations [12]. Standard management of acute rhinosinusitis typically includes pharmacological treatments such as intranasal corticosteroids, analgesics, decongestants, and, in some cases, antibiotics [13,14]. Systemic and intranasal decongestants are commonly used to provide short-term symptomatic relief, with their effectiveness widely supported by clinical use. Although generally well tolerated when used appropriately, nasal decongestants may be associated with rare but potentially serious adverse events (SAE), particularly in cases of overdose, including neurological (e.g., headache, seizures, stroke) and cardiovascular effects (e.g., hypertensive crisis, tachycardia, palpitations). Furthermore, repeated use of topical decongestants may lead to persistent or worsening nasal congestion, commonly described as “rebound congestion” or rhinitis medicamentosa, although the diagnostic criteria for these conditions remain poorly defined in clinical practice [11].

In this context, leading guidelines also emphasize the value of non-pharmacological or mechanical interventions—such as saline irrigation or barrier-based approaches—for short-term symptom relief [15]. These options are widely recommended due to their favorable safety profile and minimal risk of systemic exposure or contribution to antibiotic resistance. Consequently, there is a growing interest in therapeutic alternatives that maintain clinical efficacy while reducing systemic absorption and limiting the risk of AEs, particularly in vulnerable populations [16].

- Considerations for Pregnant Women

Pregnant individuals represent a particularly sensitive patient group in the management of rhinosinusitis. Physiological changes associated with pregnancy, including hormonal fluctuations, can contribute to increased nasal congestion. Elevated estrogen levels have been shown to increase nasal mucosal vascularization, vascular permeability, and mucosal reactivity, thereby promoting edema and nasal obstruction. Progesterone may further contribute to nasal congestion through its vasodilatory effects and its influence on plasma volume expansion. In addition, pregnancy-related factors such as increased blood volume and enhanced expression of angiogenic mediators, including vascular endothelial growth factor, may further exacerbate mucosal swelling and nasal resistance. These physiological changes can significantly impair quality of life and sleep quality, highlighting the importance of safe therapeutic approaches with minimal systemic exposure in pregnant individuals [17,18,19,20]. Consequently, several commonly used therapeutic options, such as certain decongestants and antibiotics, may be restricted or require cautious prescribing in this population. Within this context, non-pharmacological approaches offering a favorable safety profile are of particular interest. In the present study, inclusion was limited to participants in the first and second trimesters in order to reduce potential risks and to ensure appropriate monitoring of both maternal and fetal health [19,21].

- Considerations for Pediatric Populations

Children (≥3 years old) represent another important demographic requiring cautious management. While rhinosinusitis in children often resembles adult presentations, a developing immune system and potential sensitivity to certain medications underscore the importance of gentle but effective interventions [1,22]. Non-pharmacological or mechanical treatments that can reduce edema and improve mucus clearance without systemic effects are therefore particularly attractive [23,24]. In this study, parents provided consent and were given clear instructions for spray administration, while pediatricians supervised the treatment and symptom assessments, ensuring consistent and safe care.

NESOSPRAY HE-G is an internationally patented nasal device based on a filmogenic glycerol combined with plant-derived polymers. The plant-derived polymers included in the formulation are intended to interact with glycerol and contribute to the formation and stability of the protective film over the nasal mucosa. Consequently, the device is based on its physical barrier-forming and osmotic properties rather than on any direct pharmacological activity of the individual botanical constituents. This mode of action relies on two complementary mechanisms. First, the high osmotic potential promotes the mobilization of excess fluid from the inflamed mucosa, thereby contributing to a reduction in nasal obstruction and rhinorrhea. Second, the formation of a physical barrier helps limit exposure to external irritants, including pathogens and environmental factors. As the product remains confined to the nasal cavity and does not undergo systemic absorption, it acts through a localized and purely mechanical pathway. Previous clinical studies in conditions such as allergic rhinitis and sinusitis have highlighted the functional properties of this formulation, including a marked osmotic activity and its ability to establish a persistent protective layer on the nasal epithelium lasting several hours [15,25,26,27]. This characteristic supports its favorable safety and tolerability profile, making it a relevant non-pharmacological option for the management of symptoms associated with acute rhinosinusitis.

This randomized, double-blind, placebo-controlled clinical study evaluates the efficacy and safety of NESOSPRAY HE-G in relieving symptoms of acute rhinosinusitis. We recruited a broad patient population, including adults, children (aged ≥3 years), and pregnant women (in their first or second trimester), to assess the device’s performance across various subgroups while maintaining a primary focus on overall acute rhinosinusitis management. As such, the study addresses the need for safe, effective, and non-pharmacological therapies for patients who either wish to avoid or cannot tolerate standard pharmacological treatments.

## 2. Materials and Methods

### 2.1. Study Design and Ethics

The study was conducted as a randomized, double-blind, parallel-group, controlled clinical trial with a comparative design. All study procedures were performed in accordance with applicable regulatory and ethical standards, including the International Council for Harmonisation Good Clinical Practice (ICH-GCP), Indian GCP requirements, and ISO 14155 recommendations [28]. The trial was prospectively registered in the Clinical Trials Registry of India (CTRI) under the identifier CTRI/2023/02/079396 (registered on 9 June 2023). Approval of the study protocol was obtained from the relevant independent ethics committee(s) prior to study initiation. Written informed consent was obtained from all participants or, where applicable, from their legal representatives before inclusion in the study. The study was sponsored by VITROBIO SAS (France), with Polytrap Pharma Pvt. Ltd. acting as the local representative sponsor. Operational oversight and study management were carried out by Mudra Clincare in India.

### 2.2. Study Population

Acute rhinosinusitis was diagnosed by the investigator based on clinical evaluation and the presence of characteristic symptoms of ARS. Although formal application of EPOS 2020 diagnostic criteria was not required, the clinical presentation of enrolled participants was consistent with the EPOS definition of acute rhinosinusitis [12]. To ensure inclusion of patients with clinically relevant symptomatic disease, only participants presenting a baseline Rhinosinusitis Symptom Severity Score (RSSS) ≥ 25/50 were eligible for enrollment.

A comprehensive assessment of rhinosinusitis severity can be achieved using the RSSS, which integrates key symptoms such as nasal congestion, rhinorrhea, facial discomfort, cough, and impaired sleep into a single standardized score. Its composite nature captures both nasal and systemic aspects of the condition, making it well-suited for tracking clinical progression in acute and chronic rhinosinusitis [13,16]. Similar to other validated scoring tools (e.g., SNOT-22), the RSSS is designed to detect clinically meaningful changes over time, thereby enabling precise evaluation of treatment effects in both research and practice. By incorporating multiple core symptoms into a single index, the RSSS offers a sensitive means to reflect patient-reported outcomes, facilitating comparisons across various interventions while maintaining simplicity and high patient compliance.

Both adults and children were included, and pregnant women were not excluded if they met all inclusion criteria. Exclusion criteria encompassed patients with documented hypersensitivity to any of the product components, anatomical nasal abnormalities (including nasal polyps), recent nasal surgical procedures, or use of concomitant medications that could interfere with rhinosinusitis evaluation, such as antihistamines, steroids, or antibiotics administered within two weeks prior to screening. Patients unable or unwilling to comply with the study requirements, or those with immunosuppression or chronic allergy treatments, were also excluded.

### 2.3. Special Considerations for Pediatric and Pregnant Populations

For pediatric participants, inclusion in the study was contingent upon the provision of written informed consent by a parent or legal guardian. Caregivers received clear guidance on the correct administration of the product and were responsible for ensuring adherence to the treatment protocol. Throughout the study, children underwent regular follow-up by a pediatrician, who was in charge of monitoring safety parameters as well as evaluating symptom progression.

Regarding pregnant participants, eligibility was restricted to women in the first and second trimesters in order to limit potential risks. Individuals in the third trimester were not included in the study population.

### 2.4. Interventions and Randomization

Participants were assigned in a 1:1 ratio to receive either the investigational treatment (NESOSPRAY HE-G) or a placebo nasal spray. NESOSPRAY HE-G was the sole investigational product evaluated in this study and was administered to all participants allocated to the active treatment arm, including children aged ≥3 years and adults. No alternative pediatric formulation was used during clinical investigation. The allocation sequence was generated using SAS software (Version 9.1.3) with a block randomization method, ensuring balanced distribution across predefined age groups (3–18 years and >18 years). Blinding was maintained throughout the study for investigators, participants, and caregivers.

NESOSPRAY HE-G is a nasal spray formulation composed of glycerol (26%), purified water, hydroxypropylcellulose (HPC), and a complex of plant-derived polymers, including Rhinocyanidin obtained from *Camellia sinensis*, *Vaccinium myrtillus*, *Vaccinium macrocarpon*, and *Sambucus nigra*, as well as essential oils of Eucalyptus globulus, *Mentha piperita*, *Thymus*, and *Rosmarinus officinalis*. The glycerol concentration was selected during formulation development to provide an appropriate balance between osmotic activity, filmogenic capacity, physicochemical stability, and spray performance. This concentration has been used in previous formulations based on the same filmogenic technology and was considered suitable for achieving the intended mechanical mode of action of the device [29].

Its mechanism is based on a localized mechanical action combining an osmotic effect, which facilitates a reduction in mucosal edema, with the formation of a protective film over the nasal mucosa.

**Figure 1:**
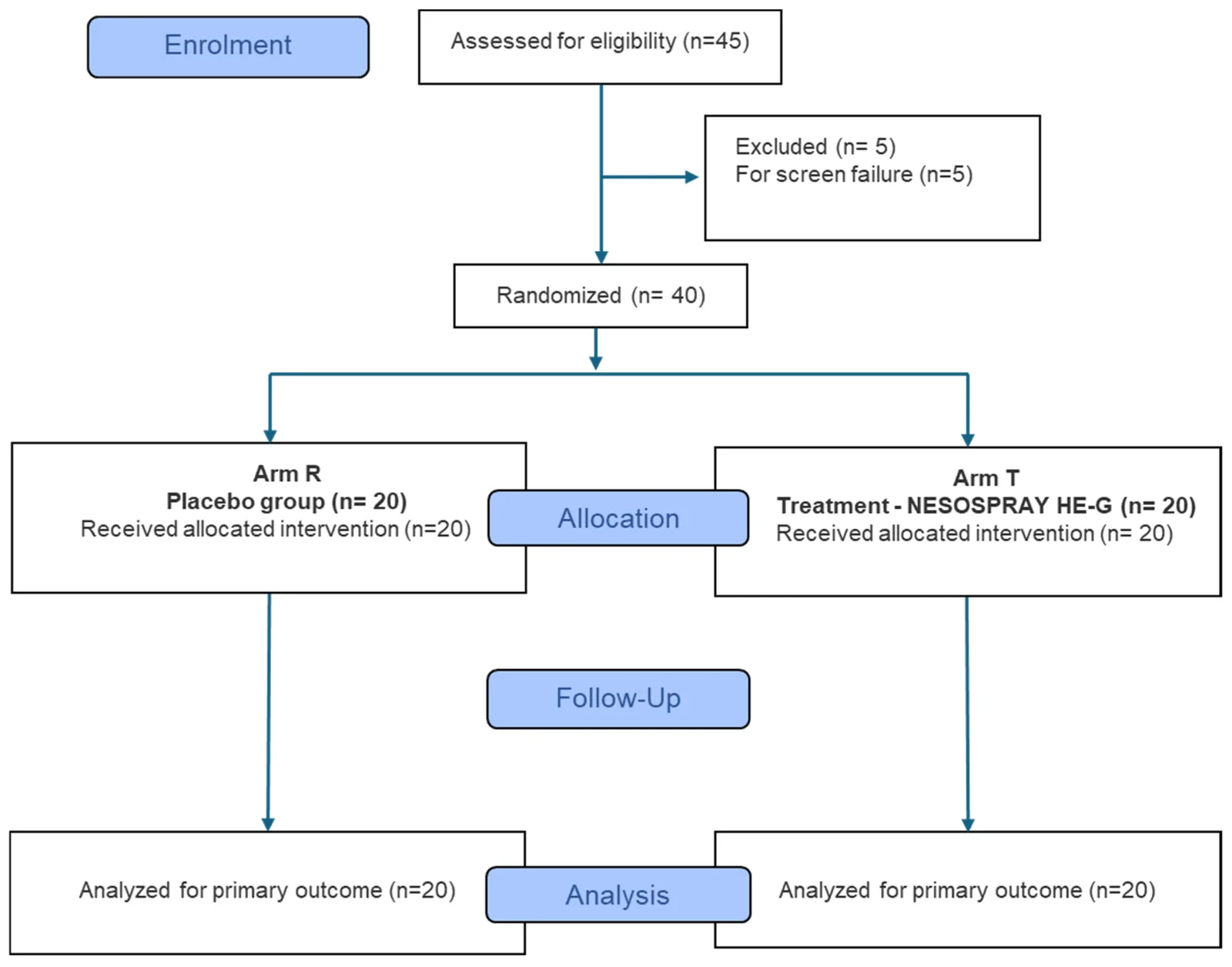
Flow diagram of participant progression through the study. Forty-five patients were screened and 5 were excluded before randomization. Forty patients were randomized and included in the analysis, and all met the inclusion criteria, with no exclusions prior to randomization. Participants were randomized in a 1:1 ratio to receive either the placebo (R, *n* = 20) or NESOSPRAY HE-G (T, *n* = 20). All randomized participants received the allocated intervention and completed the study, with no loss to follow-up or discontinuation reported in either group. All participants were included in the final analysis for the primary outcome.

The comparator consisted of a thickened aqueous solution designed to mimic the physical characteristics of the investigational product, including viscosity, visual appearance, packaging, and administration method. The formulation included solagum, potassium sorbate, purified water, sodium benzoate, and citric acid. Both treatments were administered as three sprays per nostril, four times daily (Figure 1).

### 2.5. Study Procedures and Assessments

After screening, baseline evaluations were conducted, including the collection of demographic data, vital signs, physical examination findings, and the RSSS. Follow-up assessments were scheduled at predefined time points: baseline (Day 0), 2 h after the first administration, Day 1 (24 h), Day 3, Day 6, and Day 15, or earlier in the case of symptom resolution. The follow-up schedule was designed to capture both the early onset of action of the investigational device and symptom evolution throughout the natural course of acute rhinosinusitis. The 2-h assessment was included to evaluate potential immediate effects following the first administration. Subsequent evaluations at Day 1, Day 3, and Day 6 allowed monitoring of symptom progression during the acute phase of the disease, including the period during which symptoms may worsen before recovery. The Day 15 assessment was selected to evaluate overall symptom resolution and treatment outcome, as acute rhinosinusitis is generally considered a self-limiting condition with substantial improvement occurring within approximately two weeks, in accordance with EPOS 2020 recommendations [12].

At each visit, symptom severity was recorded by the participants or, for pediatric patients, by their parents or caregivers.

The primary efficacy outcomes focused on changes in both the total RSSS and its main components, including nasal symptoms (rhinorrhea and congestion), fever, cough, sleep disturbance, and facial pain or pressure. Secondary outcomes included the evolution of individual symptom scores, overall assessments conducted by both the patient (or caregiver) and the investigator at the end of the study, as well as evaluation of product acceptability. Safety was continuously monitored throughout the study by recording AEs and SAEs.

The use of concomitant treatments, apart from the investigational or comparator products, was generally restricted. However, antibiotic therapy was permitted if considered clinically necessary by the treating physician due to worsening of the patient’s condition. Participants who received prohibited medications or failed to adhere to the study protocol were withdrawn from the trial.

### 2.6. Blinding and Unblinding

The investigational and comparator products were designed to be indistinguishable in terms of appearance, packaging, and labeling. Allocation codes were generated and maintained by authorized personnel from the sponsor, with no access provided to investigators or study staff. Blinding was preserved for all participants, investigators, and clinical personnel throughout the study period and remained in place until completion of the database lock.

In situations where patient safety required access to treatment information, unblinding could be carried out in accordance with predefined procedures. Any such instances were carefully documented and supported by appropriate justification.

### 2.7. Statistical Analysis

A total of 45 patients were enrolled, with the objective of obtaining 40 evaluable participants (20 per treatment arm). The analysis population consisted of individuals who completed all scheduled visits and assessments (Figure 1). Comparisons of baseline demographic and clinical characteristics between groups were performed using appropriate statistical methods, including Student’s *t*-test, Wilcoxon rank-sum test, and Fisher’s exact test, to verify the comparability of the study populations.

Changes in efficacy outcomes over time were assessed using repeated-measures two-way ANOVA based on mixed linear models, including Treatment, Day, and the Treatment × Day interaction. Post hoc pairwise comparisons between treatment groups at individual time points were subsequently performed using Student’s *t*-test or Wilcoxon rank-sum test, depending on data distribution. These post hoc analyses were considered supportive analyses intended to further characterize the temporal evolution of treatment effects. The incidence of AEs was evaluated using chi-square tests or Fisher’s exact test.

Although the study included a heterogeneous population (children, adults, and pregnant women), the primary statistical analyses were conducted on the overall study cohort. Additional analyses were performed to ensure that observed trends and treatment effects remained consistent across these subgroups.

## 3. Results

### 3.1. Patient Population and Baseline Characteristics

**Figure 2:**
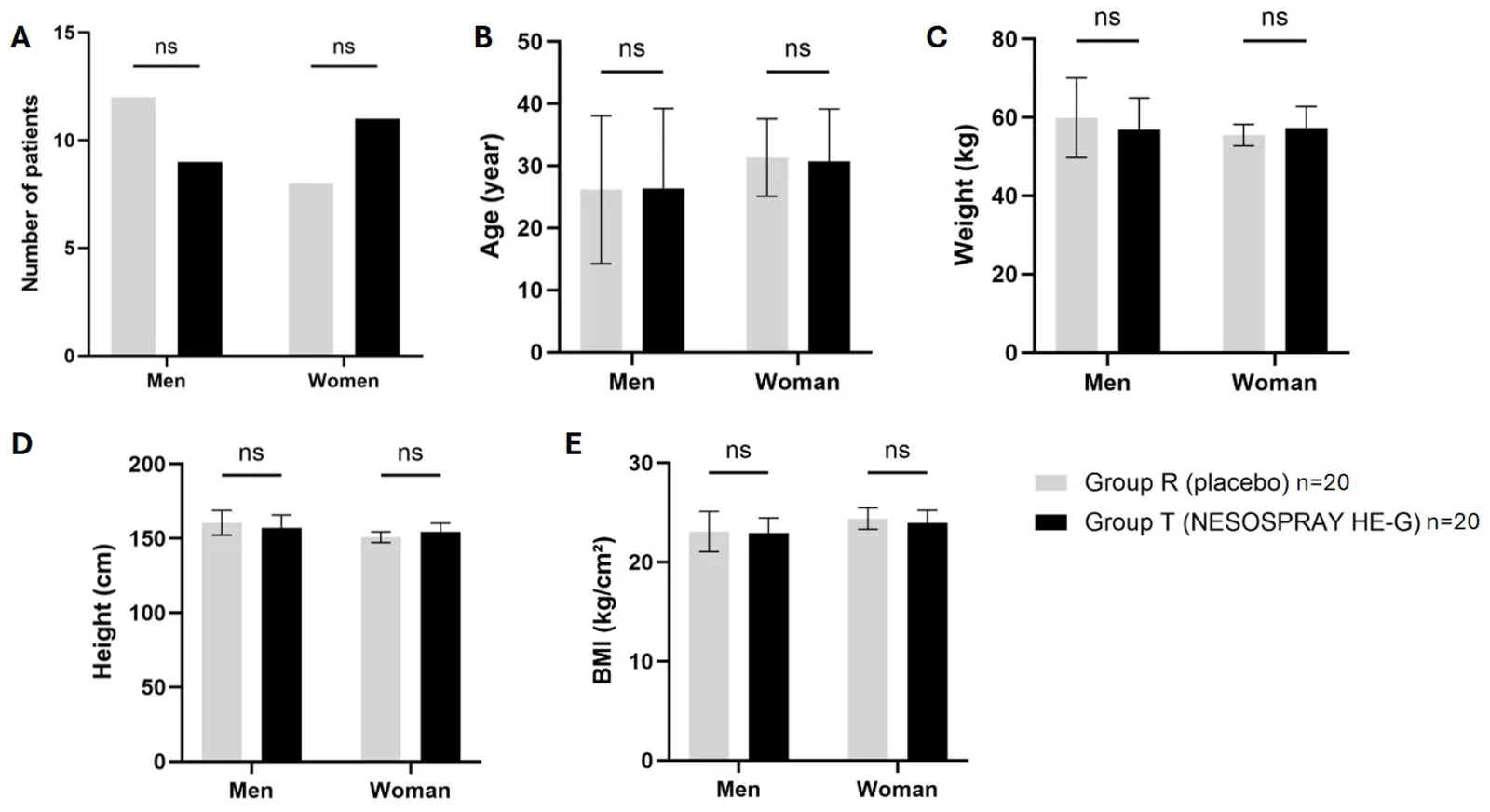
Baseline demographic and anthropometric characteristics of the study population. (**A**) Distribution of patients by sex (number of participants), (**B**) age (years), (**C**) body weight (kg), (**D**) height (cm), and (**E**) body mass index (BMI, kg/m^2^) in group R (placebo group) and group T (NESOSPRAY HE-G). Data are presented as mean ± standard deviation (SD). Sex distribution was compared using Fisher’s test, whereas continuous baseline variables (age, body weight, height, and BMI) were compared using Student’s *t*-test. No statistically significant differences were observed between the two groups within each sex category (ns: not significant).

Forty patients were included in the analysis, with 20 participants assigned to each treatment group. The distribution of participants by sex was similar between groups, with no statistically significant differences observed within each sex category. Age, body weight, height, and body mass index (BMI) were comparable between group T (NESOSPRAY HE-G) and group R (placebo) for both male and female participants. No statistically significant differences were identified for any of these parameters (all *p* > 0.05), indicating a well-balanced study population at baseline. Overall, these results confirm the comparability of the two groups in terms of key demographic and anthropometric characteristics prior to treatment initiation (Figure 1 and Figure 2).

### 3.2. Overall Symptom Severity (Primary Endpoint)

From Day 2 onwards, group T demonstrated a statistically significant reduction in the total RSSS compared to the comparator group R. This difference reached a high level of statistical significance as early as Day 2 (*p* < 1.5 × 10^−7^) and became more pronounced by Day 3 (*p* < 1.5 × 10^−12^). The superiority of NESOSPRAY HE-G was sustained over time, with significant between-group differences maintained at Day 6 (*p* < 3.0 × 10^−7^) and Day 15 (*p* < 3.1 × 10^−5^).

These results highlight both rapid onset and persistence of symptom improvement in the active treatment group. By Day 3, patients in the NESOSPRAY HE-G arm experienced a substantial reduction in RSSS, exceeding a 50% decrease from baseline on average, whereas improvements in the placebo group were more gradual and less pronounced.

**Figure 3:**
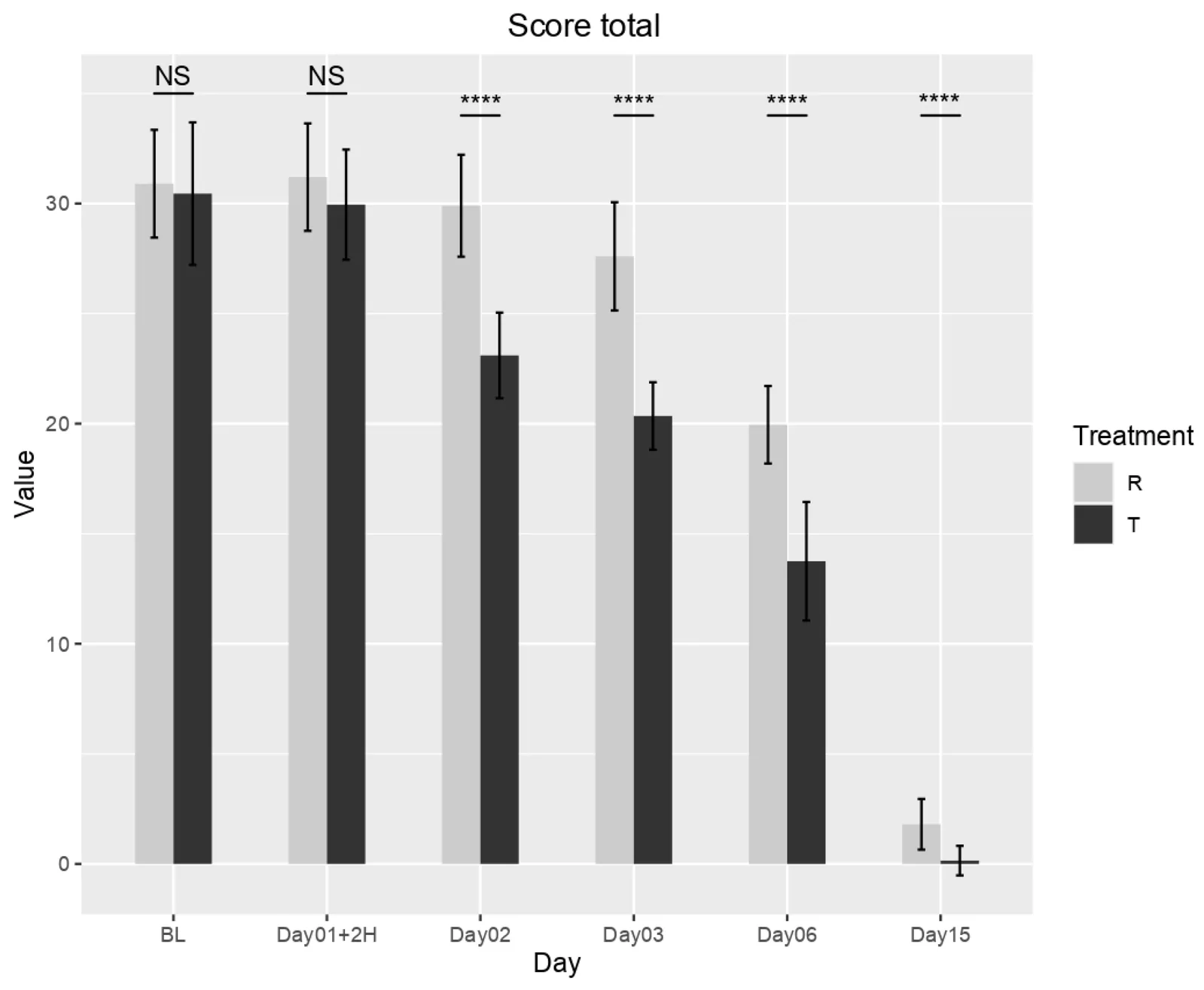
Evolution of the total RSSS over time in the NESOSPRAY HE-G and placebo groups. Mean values ± standard deviation of total RSSS are presented at baseline (BL), 2 h post-administration (Day 0–2 h), and on Days 1, 2, 3, 6, and 15, for the reference group (R, light grey) and the treatment group (T, dark grey). At BL and at early time points, no statistically significant differences were observed between groups. From Day 2 onward, a significantly greater reduction in RSSS was observed in the NESOSPRAY HE-G group compared with placebo. RSSS over time were analyzed using a two-way repeated-measures ANOVA followed by post hoc pairwise comparisons between treatment groups at each time point. NS: not significant; * Indicates statistical significance: **** *p* < 0.0001.

This divergence in both the speed and magnitude of symptom relief remained evident throughout the study period, up to Day 15 or until symptom resolution (Figure 3).

### 3.3. Individual Symptom Outcomes (Secondary Endpoints)

#### 3.3.1. Nasal Congestion (Rhinorrhea/Congestion)

No significant difference in nasal congestion was observed between groups at baseline (*p* = 0.68). However, a divergence between treatments became apparent as early as 2 h following the first administration (*p* = 0.0407), favoring the NESOSPRAY HE-G group. This difference increased over time and remained statistically significant at all subsequent evaluations, including Day 2 (*p* < 4.2 × 10^−7^), Day 3 (*p* < 3.4 × 10^−7^), Day 6 (*p* < 7.2 × 10^−5^), and Day 15 (*p* < 1.8 × 10^−5^).

**Figure 4:**
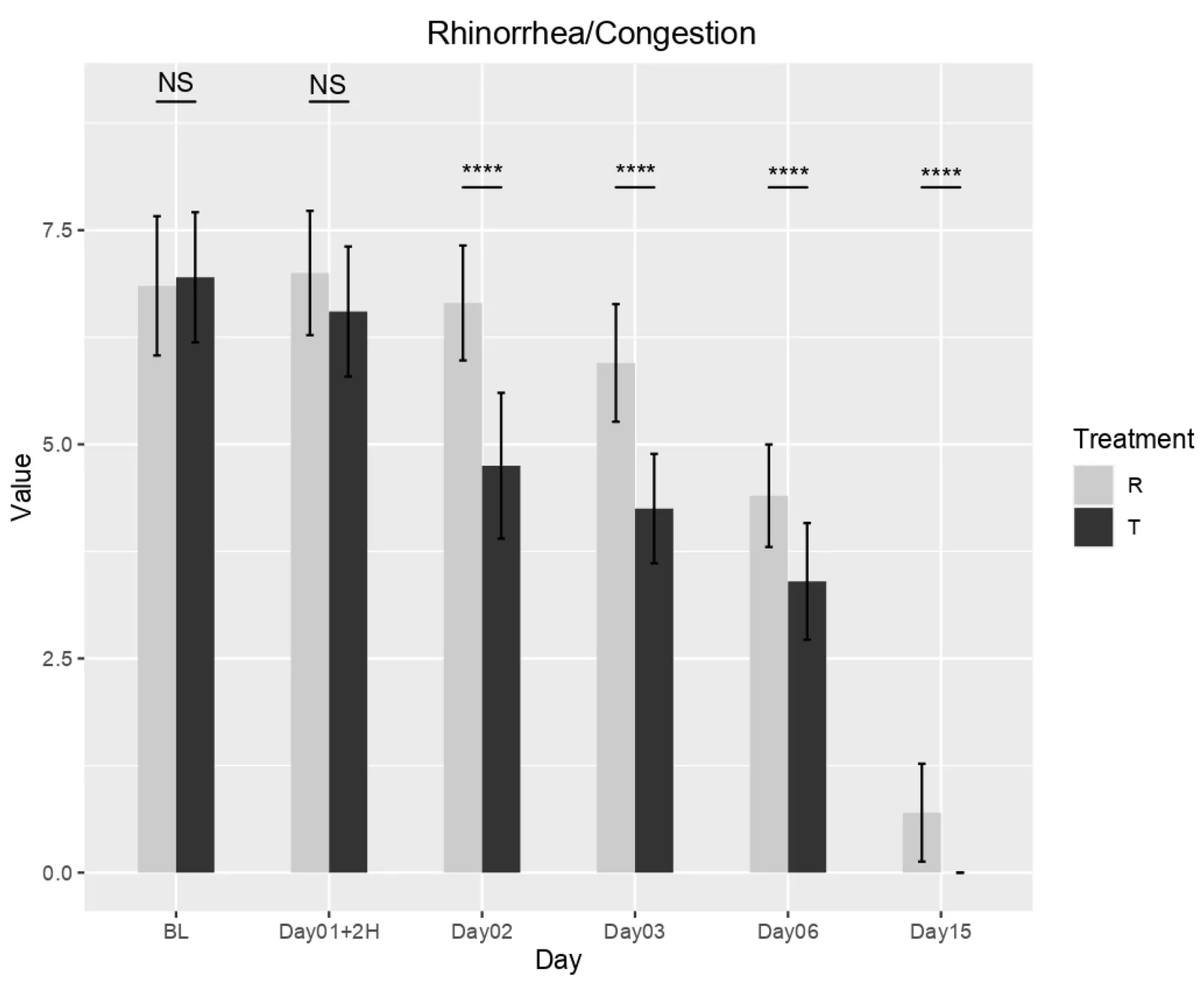
Evolution of rhinorrhea/congestion scores over time. Mean values (±standard deviation) of rhinorrhea/congestion scores are presented for the reference group (R, light grey) and the treatment group (T, dark grey) at baseline (BL), Day 0 + 2 h, Day 2, Day 3, Day 6, and Day 15. Both groups show a progressive decrease in symptom scores over time, with a greater reduction observed in the treatment group from Day 2 onwards. Rhinorrhea/congestion over time was analyzed using a two-way repeated-measures ANOVA followed by post hoc pairwise comparisons between treatment groups at each time point. Data are presented as mean ± SD. NS: not significant; statistical significance: **** *p* < 0.0001.

Patients treated with NESOSPRAY HE-G showed a faster and more pronounced decline in nasal congestion scores compared with the placebo group, which was associated with improved nasal airflow and overall comfort. These effects were sustained throughout the study period (Figure 4 and Table 1).

#### 3.3.2. Cough

At study entry, cough scores were comparable between the two groups (*p* = 0.56), and no significant difference was observed following the first administration at 2 h (*p* > 0.2). A clear separation between treatment arms became evident from Day 2, with patients receiving NESOSPRAY HE-G showing significantly lower cough scores (*p* < 1.8 × 10^−5^). This between-group difference was maintained at all subsequent time points, including Day 3 (*p* < 1.4 × 10^−5^), Day 6 (*p* < 2.9 × 10^−6^), and Day 15 (*p* = 0.02).

**Figure 5:**
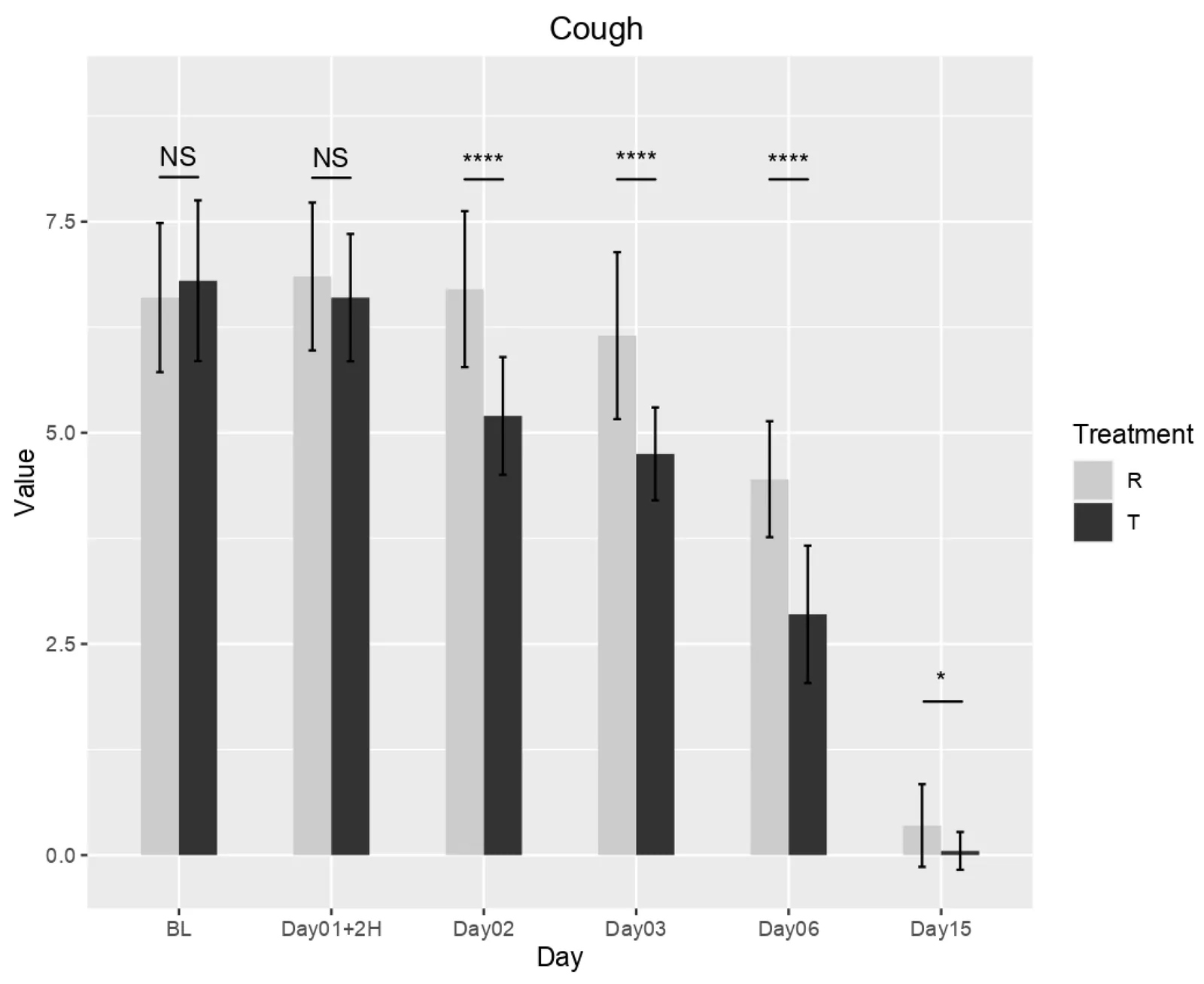
Evolution of cough scores over time. Mean cough scores (± standard deviation) are presented for the reference group (R, light grey) and the treatment group (T, dark grey) at baseline (BL), Day 0 + 2 h, Day 2, Day 3, Day 6, and Day 15. Both groups exhibited a progressive reduction in cough severity over time. A greater decrease was observed in the treatment group from Day 2 onwards, with statistically significant differences between groups at multiple time points. Cough over time was analyzed using a two-way repeated-measures ANOVA followed by post hoc pairwise comparisons between treatment groups at each time point. NS: not significant; statistical significance: * *p* < 0.05; **** *p* < 0.0001.

Overall, treatment with NESOSPRAY HE-G was associated with a consistent reduction in cough severity and/or frequency compared with placebo, with effects sustained throughout the study period (Figure 5 and Table 1).

#### 3.3.3. Sleep Disturbance

**Figure 6:**
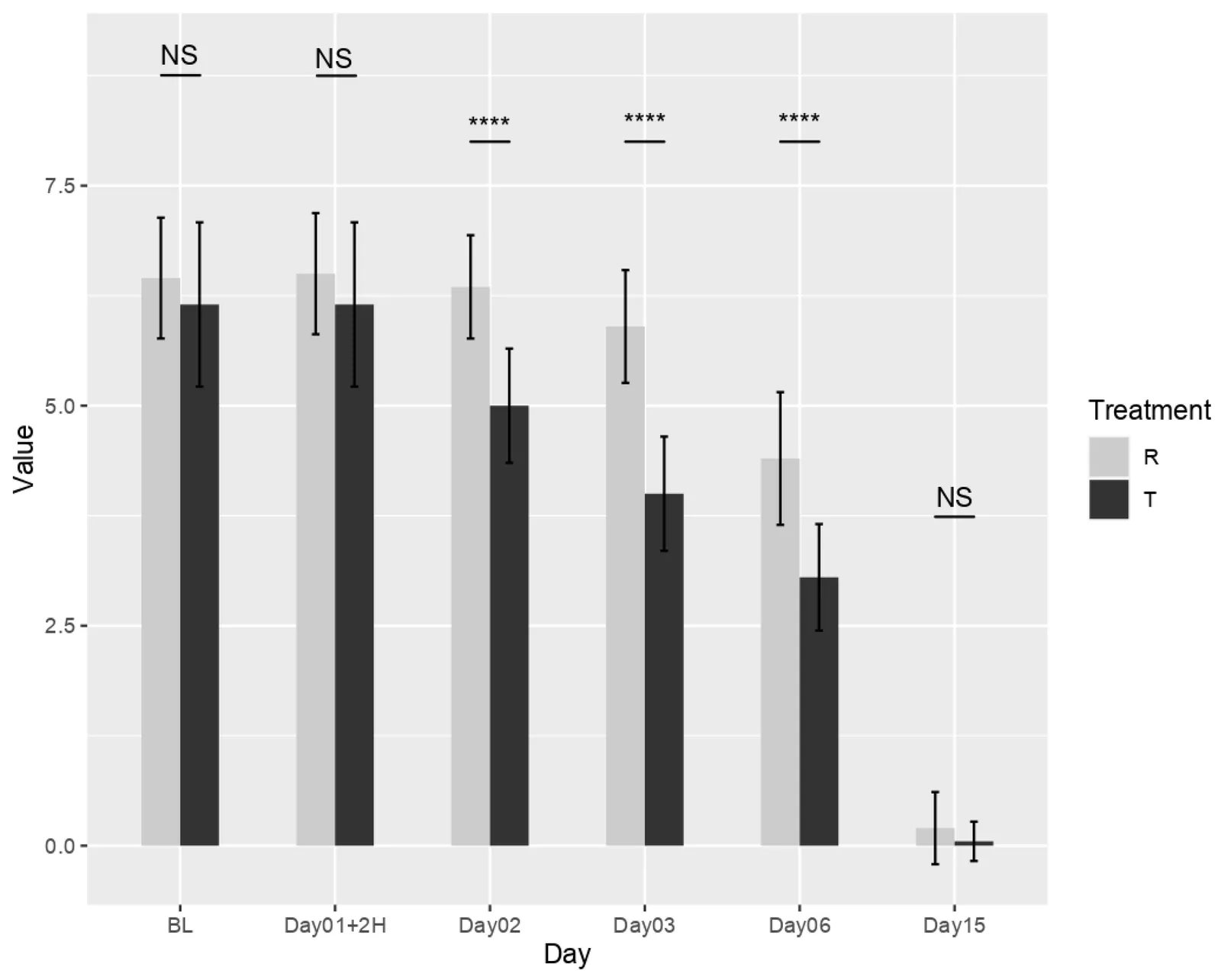
Evolution of sleep disturbance scores over time. Mean scores (±standard deviation) for sleep disturbance are presented for the reference group (R, light grey) and the treatment group (T, dark grey) at baseline (BL), Day 0 + 2 h, Day 2, Day 3, Day 6, and Day 15. Both groups showed a progressive reduction in sleep disturbance over time. A more pronounced improvement was observed in the treatment group from Day 2 to Day 6, with statistically significant differences between groups at these time points. No significant differences were observed at baseline, Day 0 + 2 h, and Day 15. Sleep disturbance over time was analyzed using a two-way repeated-measures ANOVA followed by post hoc pairwise comparisons between treatment groups at each time point. NS: not significant; * Indicates statistical significance: **** *p* < 0.0001.

Improvements in sleep quality followed a similar pattern. While baseline differences were not significant (*p* > 0.2), by Day 2 a highly significant benefit emerged for NESOSPRAY HE-G (*p* < 1.9 × 10^−6^), remained at Day 3 (*p* < 1.2 × 10^−7^) and Day 6 (*p* < 5.1 × 10^−6^). By Day 15, sleep scores converged between groups (*p* = 0.16), likely due to natural symptom resolution over time (Figure 6 and Table 1).

#### 3.3.4. Pain upon Facial Pressure

**Figure 7:**
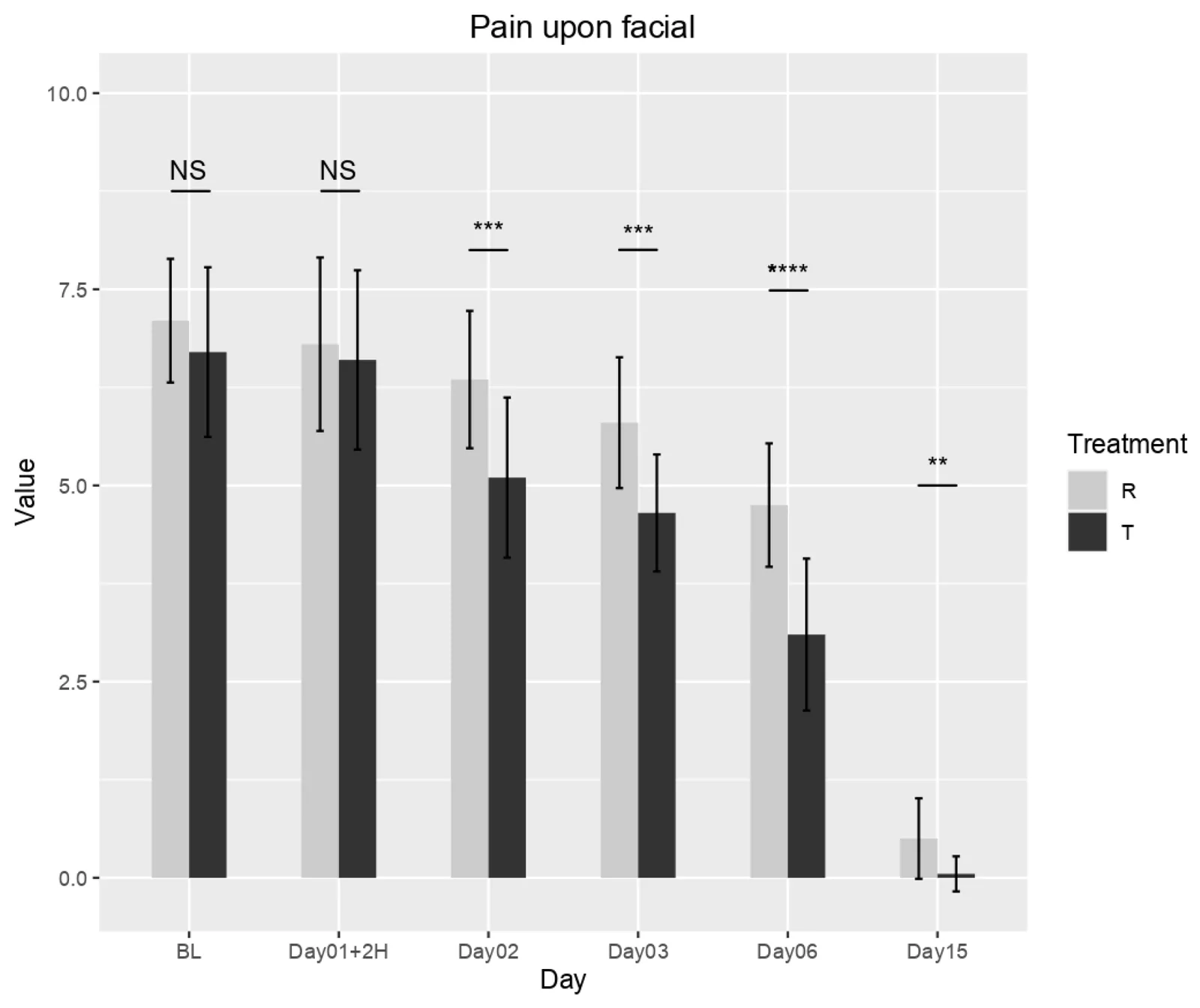
Evolution of facial pain scores over time by treatment group. Mean facial pain scores (±standard deviation) are presented for the reference group (R, light grey) and the treatment group (T, dark grey) at baseline (BL), Day 0 + 2 h, Day 2, Day 3, Day 6, and Day 15. Both groups showed a progressive decrease in facial pain over time. A greater reduction was observed in the treatment group from Day 2 onwards, with statistically significant differences between groups at several time points. No significant differences were observed at baseline and Day 0 + 2 h, while significant differences were observed at Day 2 (***), Day 3 (***), Day 6 (****), and Day 15 (**). Facial pain over time was analyzed using a two-way repeated-measures ANOVA followed by post hoc pairwise comparisons between treatment groups at each time point. NS: not significant; * Indicates statistical significance: ** *p* < 0.01; *** *p* < 0.001; **** *p* < 0.0001.

Pain scores did not differ initially (*p* = 0.25), but NESOSPRAY HE-G led to significant reductions starting Day 2 (*p* < 3.4 × 10^−4^), sustained through Day 3 (*p* < 1.6 × 10^−4^), Day 6 (*p* < 8.6 × 10^−6^), and Day 15 (*p* < 0.002). This continued improvement indicates effective relief of sinus-related pressure and discomfort (Figure 7 and Table 1).

#### 3.3.5. Fever

**Figure 8:**
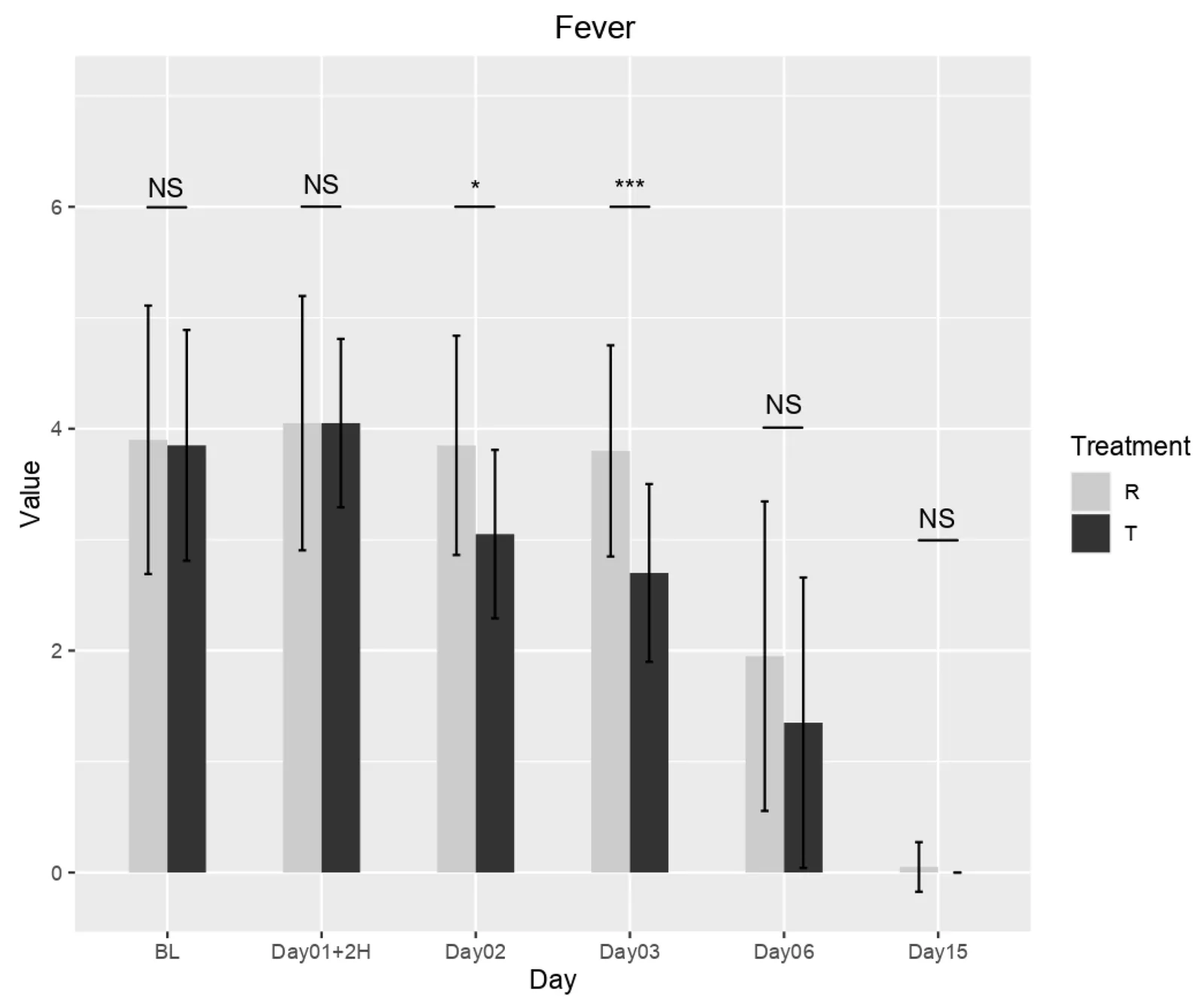
Evolution of fever scores over time by treatment group. Mean fever scores (±standard deviation) are presented for the reference group (R, light grey) and the treatment group (T, dark grey) at baseline (BL), Day 0 + 2 h, Day 2, Day 3, Day 6, and Day 15. Both groups showed a progressive reduction in fever over time. A greater decrease was observed in the treatment group at Day 2 and Day 3, with statistically significant differences between groups at these time points. No significant differences were observed at baseline, Day 0 + 2 h, Day 6, and Day 15. Fever score over time was analyzed using a two-way repeated-measures ANOVA followed by post hoc pairwise comparisons between treatment groups at each time point. NS: not significant; statistical significance: * *p* < 0.05; *** *p* < 0.001.

Both groups saw fever resolution over time, but NESOSPRAY HE-G provided an earlier improvement. Differences reached significance at Day 2 (*p* = 0.01) and Day 3 (*p* = 8.1 × 10^−4^) before diminishing by Day 6 (*p* = 0.18) and Day 15 (*p* = 0.34), as most patients had recovered regardless of treatment allocation (Figure 8 and Table 1).

### 3.4. Global Assessments

At the end of the study (Day 15), overall evaluations performed by patients (or caregivers) and investigators descriptively suggested a more favorable outcome in the NESOSPRAY HE-G group compared with placebo. A higher proportion of participants in the active treatment arm were classified as showing marked or substantial improvement. These assessments were collected as supportive descriptive outcomes and were not predefined as formal efficacy endpoints. Consequently, no statistical comparison was performed, and the results should be interpreted as complementary observations supporting the overall clinical findings.

### 3.5. Antibiotic Use

Investigators were permitted to prescribe antibiotics when considered clinically necessary because of symptom worsening. During the study period, antibiotic treatment was required in 15 of 20 participants in the placebo group compared with only 1 of 20 participants in the NESOSPRAY HE-G group.

### 3.6. Safety and Tolerability

Both groups were generally well tolerated throughout the study, with no SAEs reported. The incidence of mild AEs, including local irritation, was similar between groups, with no statistically significant difference observed (*p* > 0.7). No allergic reactions were recorded during the study. Overall, the safety profile remained consistent across all age categories, including pregnant participants.

### 3.7. Subgroup Consistency

Although the study enrolled a heterogeneous population including children (≥3 years), adults, and pregnant women, efficacy was not formally analyzed in separate subgroup-specific models. Instead, the consistency of the treatment response across subpopulations was explored by assessing the interaction between treatment assignment and subgroup within the repeated-measures framework used for the overall study population.

No significant treatment-by-subgroup interaction was identified, suggesting that the pattern of clinical improvement observed with NESOSPRAY HE-G was comparable across the different participant categories. In particular, pregnant women included in the study—limited to the first and second trimesters, with no third-trimester participants—showed a magnitude and timing of symptom improvement similar to those observed in non-pregnant adults (interaction *p* > 0.05). Likewise, children aged 3 years and older followed a symptom resolution profile comparable to that seen in the overall population, indicating that age did not appear to influence the treatment response (interaction *p* > 0.05).

This overall consistency was also supported by the safety data, which did not reveal any subgroup-specific concerns. The frequency of AEs and the general tolerability profile were similar across age categories and among pregnant participants, in line with the findings observed in the total study cohort. Taken together, these results support the view that NESOSPRAY HE-G maintains a consistent efficacy and safety profile in children, adults, and pregnant women, despite the absence of formal subgroup-dedicated analyses.

## 4. Discussion

This randomized, double-blind, placebo-controlled trial highlights the clinical efficacy of NESOSPRAY HE-G, a non-pharmacological nasal medical device composed of a filmogenic glycerol formulation associated with plant-derived polymers. These botanical components are primarily intended to interact with glycerol and contribute to the formation and stabilization of the protective film on the nasal mucosa, thereby supporting the device’s mechanical mode of action. Compared with placebo, patients receiving NESOSPRAY HE-G experienced both early and sustained symptom improvement.

A significant reduction in key symptoms, including nasal congestion, facial pain, cough, and impaired sleep, was observed as early as Day 2. Notably, an initial effect on nasal congestion could already be detected within hours following administration. The magnitude of improvement continued to increase over time and remained evident through Day 15, supporting a durable treatment effect. An additional clinically relevant observation was the lower use of antibiotics among participants treated with NESOSPRAY HE-G compared with placebo. Although this outcome was not a predefined endpoint, the finding may warrant further investigation in future studies. These clinical outcomes are consistent with the proposed mechanism of action of NESOSPRAY HE-G. Its osmotic properties are expected to promote fluid clearance from the edematous nasal mucosa, thereby reducing congestion, while the formation of a protective film may limit ongoing irritation from environmental factors or pathogens. Together, these effects are likely to contribute to both the rapid onset and persistence of symptom relief observed in this study.

The results align with previous clinical recommendations advocating for non-pharmacological approaches in managing rhinosinusitis, especially in patient populations where drug-based therapies may pose risks or be less tolerated. Traditional pharmacotherapy, including intranasal corticosteroids and antibiotics, can be effective but raises concerns regarding systemic side effects, antibiotic resistance, and contraindications in certain patient groups [12,14,30].

The consistent efficacy observed across different patient groups, including children and pregnant women, suggests that NESOSPRAY HE-G may be suitable for use in a broad clinical population. The pediatric population included participants across a broad age range (3–18 years), encompassing different stages of physiological maturation, including pubertal development. Although age-related differences in sinonasal anatomy, immune maturation, and symptom perception may contribute to variability in disease presentation, the investigational device acts locally through a mechanical mode of action without systemic absorption [31,32,33]. Therefore, a major influence of developmental status on the mechanism of action of the device is not expected. Nevertheless, pubertal status was not specifically recorded and may be considered in future studies to further characterize pediatric subgroups. However, its favorable safety profile, characterized by the absence of SAEs and allergic reactions, further supports its potential role as either a first-line or complementary therapeutic option. Compared with conventional approaches such as saline irrigation, the combined osmotic and barrier-based mechanism of NESOSPRAY HE-G could offer additional clinical benefit. Nevertheless, direct comparative studies with other non-pharmacological interventions would be valuable to better position this device within current treatment strategies.

The present findings are consistent with previous studies evaluating filmogenic glycerol-based nasal devices, which reported improvements in symptoms associated with upper respiratory tract conditions, including rhinosinusitis and rhinopharyngitis [15,29]. Clinical benefits have been reported with saline-based nasal irrigation devices and sodium hyaluronate-containing nasal formulations, which have demonstrated improvements in sinonasal symptoms, nasal comfort, and olfactory function through local, non-pharmacological mechanisms acting directly on the nasal mucosa [34,35,36]. Similarly, participants treated with NESOSPRAY HE-G experienced significant improvements in overall symptom burden, including rhinorrhea/congestion, headache, sleep disturbance, and global assessments compared with placebo. Although direct comparisons between studies should be interpreted with caution because of differences in study design, patient populations, and outcome measures, the consistency of these findings supports the clinical value of locally acting medical devices in the symptomatic management of acute rhinosinusitis. The observed benefits are in line with the therapeutic rationale of non-pharmacological nasal interventions, which aim to improve mucosal conditions, facilitate nasal function, and promote symptom relief without systemic pharmacological exposure.

Despite these encouraging results, several limitations should be acknowledged. The study included a relatively limited number of participants, the repeated-measures design enabled multiple assessments over time and allowed evaluation of both treatment and temporal effects through mixed-model analysis. Nevertheless, confirmation of these findings in larger adequately powered studies would further strengthen the evidence supporting the clinical performance of the device. Further studies with extended follow-up would be useful to better understand the duration of the observed benefits. In addition, such investigations would be particularly relevant in the context of CRS. In addition, the study population included children, adults, and pregnant women, thereby reflecting a broad range of individuals affected by acute rhinosinusitis. Although this enhances the general applicability of the findings, the study was not specifically powered to evaluate efficacy separately within each subgroup. Because the investigational device acts locally through a mechanical mode of action without systemic absorption, substantial differences in treatment response related to age or pregnancy status are not anticipated. Nevertheless, subgroup-specific analyses may provide additional insights and could be considered in future clinical investigations. Future studies could also investigate the performance of NESOSPRAY HE-G under different environmental conditions and across various etiologies of rhinosinusitis. Another limitation of the present study is the reliance on symptom-based clinical outcomes without inclusion of additional validated patient-reported outcome measures or objective assessments of nasal function. The RSSS was selected because it specifically captures the major symptoms associated with acute rhinosinusitis and enables evaluation of short-term symptom evolution during the active phase of disease. Nevertheless, future studies could benefit from incorporating complementary assessments, such as the SNOT-22, peak nasal inspiratory flow, rhinomanometry, or endoscopic evaluations, to provide a more comprehensive characterization of treatment effects [12].

Despite these considerations, the present findings provide strong evidence supporting the rapid onset of action and sustained symptom improvement associated with NESOSPRAY HE-G. Altogether, these results reinforce its potential as a safe, effective, and broadly applicable non-pharmacological option for the management of rhinosinusitis.

## 5. Conclusions

Taken together, the results of this study highlight the relevance of a non-pharmacological, mechanically driven approach in the management of acute rhinosinusitis. The observed pattern of symptom evolution, characterized by early improvements and sustained benefits over time, suggests that NESOSPRAY HE-G may effectively address key manifestations of the condition in a clinically meaningful manner. In addition, the favorable tolerability profile and the absence of safety concerns across the studied population—including children and pregnant women—support its use in patient groups for whom therapeutic options may be limited. Rather than replacing existing treatments, NESOSPRAY HE-G may be considered as a complementary or alternative strategy within current care practices, particularly when minimizing systemic exposure is desirable. Overall, these findings contribute to the growing interest in non-pharmacological interventions for upper respiratory conditions and support further exploration of their role in routine clinical management.

## Figures and Tables

**Table 1:** Summary of Statistical Tests for Different Days and Treatments. *p*-values comparing treatment groups (NESOSPRAY HE-G versus placebo) were calculated for each symptom domain—rhinorrhea/congestion, cough, sleep disturbance, facial pain, and fever—at all assessment time points (baseline, 2 h, Day 2, Day 3, Day 6, and Day 15). At baseline and shortly after administration (2 h), no statistically significant differences were observed between groups (ns). However, from Day 2 onward, significant between-group differences emerged for several symptoms, particularly rhinorrhea/congestion, cough, sleep disturbance, and facial pain, indicating greater improvement in the NESOSPRAY HE-G group. Highly significant *p*-values (*** or ****) were consistently reported for these domains at later time points. In contrast, fever did not show a consistent pattern of significant differences between groups throughout the study period. Overall treatment effects over time were analyzed using a two-way repeated-measures ANOVA followed by post hoc pairwise comparisons between treatment groups at each time point. NS: not significant; statistical significance: * *p* < 0.05; ** *p* < 0.01; *** *p* < 0.001; **** *p* < 0.0001.

Symptoms	Rhinorrhea/ Congestion	Cough	Sleep	Facial Pain	Fever
Baseline	0.6755 (NS)	0.5592 (NS)	0.2988 (NS)	0.2510 (NS)	0.9554 (NS)
After 2 h	0.0407 (*)	0.2408 (NS)	0.2199 (NS)	0.5546 (NS)	0.8647 (NS)
Day 02	4.19 × 10^−7^ (****)	1.76 × 10^−5^ (****)	1.90 × 10^−6^ (****)	3.38 × 10^−4^ (***)	0.0101 (*)
Day 03	3.35 × 10^−7^ (****)	1.33 × 10^−5^ (****)	1.13 × 10^−7^ (****)	1.58 × 10^−4^ (***)	8.09 × 10^−4^ (***)
Day 06	7.20 × 10^−5^ (****)	2.82 × 10^−6^ (****)	5.03 × 10^−6^ (****)	8.56 × 10^−6^ (****)	0.1847 (NS)
Day 15	1.76 × 10^−5^ (****)	0.0202 (*)	0.1637 (NS)	0.0018 (**)	0.3421 (NS)

## Data Availability

The original contributions presented in this study are included in the article. Further inquiries can be directed to the corresponding author.

## Abbreviations

The following abbreviations are used in this manuscript:

AE	Adverse Event
ANOVA	Analysis of Variance
BL	Baseline
BMI	Body Mass Index
CC BY	Creative Commons Attribution
CRS	Chronic Rhinosinusitis
CTRI	Clinical Trials Registry of India
GCP	Good Clinical Practice
HPC	Hydroxypropylcellulose
ICH	International Council for Harmonisation
NHG	NESOSPRAY HE-G
NS	Not Significant
RSSS	Rhino-Sinusitis Severity Score
SAE	Serious Adverse Event
SAS	Statistical Analysis System
SNOT-22	Sinonasal Outcome Test-22
